# Research Note: Evaluation of microbial contamination on broiler carcasses – an investigation of body parts and sampling methods

**DOI:** 10.1016/j.psj.2025.105538

**Published:** 2025-07-05

**Authors:** Bastian Wyink, Alina Kirse, Felix Reich

**Affiliations:** a Frankenförder Forschungsgesellschaft mbH (FFG), Luckenwalde, Germany; b Institute of Biometry, Epidemiology and Information Processing, WHO Collaborating Centre for Research and Training for Health at the Human-Animal-Environment Interface, University of Veterinary Medicine, Hannover, Germany; c German Federal Institute for Risk Assessment, Berlin, Germany

**Keywords:** broiler carcass, neck skin sampling, total viable count, *E. coli, Campylobacter*

## Abstract

We investigated whether samples of the neck skin from 24 broiler chickens, which were taken from broiler carcasses after chilling, are representative for the microbiological load (TVC – total viable count 3.99 log_10_ cfu/g, *E. coli* 2.06 log_10_ cfu/g, *Campylobacter* spp. 2.04 log_10_ cfu/g) of the entire skin. No significant difference was found between the contamination of the neck skin and the other skin areas analysed (back, breast, abdominal and leg skin) for any of the target germs. We also compared results from two sampling methods (neck skin excision and whole carcass rinse) to check whether both methods yield comparable results. Significantly higher mean values were found for the TVC in the whole carcass rinse samples (4.61 log_10_ cfu/ml vs. 4.28 log_10_ cfu/g), while we found no significant difference between both sampling methods for *E. coli*. For *Campylobacter* spp. the neck skin samples showed significantly higher mean values than the whole carcass rinse samples (2.04 log_10_ cfu/g vs. 1.31 log_10_ cfu/ml).

## Introduction

Several microbial indicators are used to monitor process hygiene in slaughterhouses. The total viable count (TVC) is often employed to assess general process hygiene, while *Escherichia coli (E. coli)* can be used in poultry slaughterhouses to account for faecal contamination (EFSA, 2012). The analysis of the two indicators is voluntary as there is no legal requirement to date. High faecal contamination can indicate the presence of zoonotic pathogens such as *Campylobacter* spp., which is the most important pathogen causing foodborne zoonoses in the European Union (EU) (EFSA, 2012). Since poultry meat is considered to be the main source of campylobacteriosis, monitoring and reducing the contamination of food with *Campylobacter* spp. are important steps towards preventing human infections. *Escherichia coli* is often discussed as a process hygiene indicator with the advantages of lower costs, easier counting and the more frequent occurrence, which unlike *Campylobacter* spp. is not affected by seasonal fluctuations (EFSA, 2012). However, the sampling methods used for assessing process hygiene differ not only between countries, but also in some cases between individual slaughterhouses (Nagel Gravning et al., 2021). The success of such monitoring is therefore influenced by the type and accuracy of the sampling method. In the European Union neck skin sampling for *Campylobacter* enumeration is mandatory for assessing process hygiene according to the Commission Regulation (EC) 2073/2005. An alternative sampling method is the whole carcass rinse (WCR), which is often used in scientific research (Nagel Gravning et al., 2021). Neck skin sampling is convenient as there is no value loss in the carcass and anecdotal reports indicate that neck skin is the most contaminated area of a broiler carcass during slaughtering and therefore the most appropriate sampling area for carcass contamination testing.

In the view of the aforementioned aspects in the present study, broiler carcasses were tested for contamination levels in various areas of the carcass skin. Skin excision was applied to determine the TVC and the contamination with *E. coli* and *Campylobacter* spp. Contamination levels on skin samples were compared with the results of neck skin samples to check whether neck skin samples are representative for the contamination of the entire carcass. We also compared the results form sampling methods of neck skin excision to whole carcass rinse data from a previous study (Beterams et al., 2024a).

## Materials and methods

### Sampling

The samples were taken between July and November 2022 in a broiler slaughterhouse in Germany. A total of 24 broiler carcasses were collected from the slaughter line after air chilling. The cooling system was a combination of pre-cooling in a water bath and final cooling in cold air. A carcass temperature of 2.5 – 3°C was reached within 140 min. Samples were taken on seven different dates. On each date, 3-4 carcasses of one flock were sampled, which in advance had been tested positive for *Campylobacter* ssp. by the slaughterhouse operator. The carcasses were placed in sterile plastic bags (400×500 mm, PA/PE 90, sealed bag, HEIFO GmbH & Co. KG, Osnabrück, Germany) and transported to the laboratory refrigerated at 4°C within a few hours. In the laboratory, individual skin sections were prepared using sterile tweezers and scalpel. We prepared six different skin areas on each carcass (neck, back, breast, abdomen, left thigh and right thigh). The wing skin could not be removed cleanly and was therefore not included in the investigations of this study. For the comparison of the two sampling methods, we used the above-mentioned neck skin samples from 24 carcasses and whole carcass rinse (WCR) samples which were both taken from the same flocks. Data for WCR - whole of 85 broilers from a previous study (Beterams et al., 2024a) was included. Broilers were taken from the slaughter-line after chilling and transferred into sterile plastic bags (400×500 mm, PA/PE 90, sealed bag, HEIFO GmbH & Co. KG) and rinsed with 400 ml of fluid (sodium chloride 0.85 %, peptone 0.1 %, 1.12535, Merck, Darmstadt, Germany, 0.75 g/l agar, LP0011, OXOID, Wesel, Germany), as described in ISO 17604:2015. The carcass was rinsed inside and outside by shaking the bag for 60 s. Every five seconds the bag was rotated 180 °. Afterwards, 200 ml of the rinsing fluid was transferred into sterile plastic cups (300 ml, PP, VWR International GmbH, Darmstadt, Germany) and transported to the laboratory refrigerated at 4°C.

### Microbiological analysis

From each skin area 10 g of skin were individually diluted 1:10 with buffered peptone water (Merck) and blended for 60 s (ISO 6887-1:2017). Tenfold dilutions were prepared for skin and WCR samples for further analyses. TVC and generic *E. coli* (hygienic indicator) counts were determined by plating 1 ml of the 10-fold dilutions into a petri dish doused with plate count agar (PC, MAST Diagnostika GmbH, Reinfeld, Germany) for TVC and tryptin-bile-X-glucuronide agar (TBX, Merck) for *E. coli*. Plate count agar was incubated for 72 h at 30°C aerobically (ISO 4833-2:2022), while TBX agar was incubated at 44°C for 24 h aerobically (ISO 16649-3:2018). The bacterial load of *Campylobacter* was determined by streaking 1 ml of the 1:10 dilution on three plates of mCCD-agar (OXOID) and incubation them for 48 h at 41.5°C under microaerobic conditions (ISO 10272-2:2017). Furthermore 0.1 ml of each dilution was streaked on mCCD-agar incubated as described before. Suspicious colonies were counted and up to three colonies per morphology were picked and streaked on non-selective Columbia blood agar (OXOID) and incubated at 41.5°C for 48 h under microaerobic conditions. These colonies were used for confirmation by microscopy (typical motility and morphology) and oxidase tests (Oxidase strips for microbiology, SIGMA ALDRICH, Taufkirchen, Germany). Detection limits for TVC, *E. coli* and *Campylobacter* were 10 CFU/g.

### Statistical analysis

The bacterial count data were log_10_ transformed. Statistical analyses were conducted in SAS (version 9.4, SAS Institute Inc., Cary, USA). A one-factor ANOVA was carried out to compare the bacterial load of the individual skin areas (*N* = 24). Multiple mean comparisons with Tukey-Kramer correction were conducted as post-hoc tests. The results of the neck skin samples and the WCR samples were tested for normal distribution and compared using an independent two-sample t-test. The choice of test was depending on the variance homogeneity of the samples. If variance homogeneity was present, the pooled t-test was used and if heterogeneity was present, the Welch-Satterthwaite test was used. The level of significance for all analyses was set to α = 0.05.

## Results and discussion

### Comparison of contamination on skin samples

**Fig. 1:**
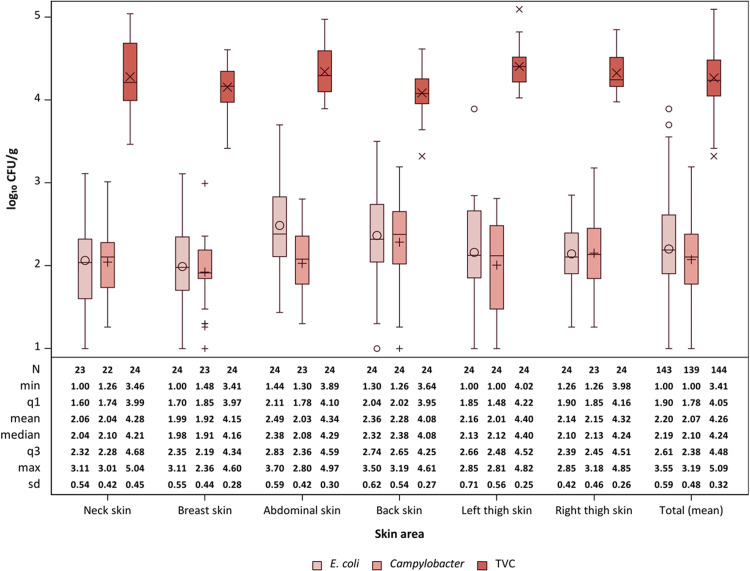
Comparison of bacterial loads (log_10_ CFU/g) of broiler skin samples for TVC, E. coli and Campylobacter taken after chilling. N: number of samples, min: minimum value, q1: first quartile, q3: third quartile, max: maximum value, standard deviation, Thigh skin l.: left thigh skin, Thigh skin r.: right thigh skin.

The contamination level on different areas of skin on broiler carcasses after chilling was analysed for 24 carcasses. We could not find a fundamental difference in the contamination levels of the examined skin areas from broiler carcasses for the tested bacteria The overall mean for all samples of the TVC on the broiler skin was 4.26 ± 0.19 log_10_ CFU/g (Fig. 1). We found that the TVC load of the back skin was lower (*P* = 0.006) than the skin of the left thigh. The TVC mean of the back skin and the abdominal skin were just barely not different (*P* = 0.052). There was no difference (*P* > 0.05) in TVC loads between neck skin and any other examined skin area. For *E. coli* the overall mean was 2.21 ± 0.42 log_10_ CFU/g (Fig. 1). We found no difference (*P* > 0.05) between the neck skin and any other sampled skin area for *E. coli* contamination. The comparison of *E. coli* contamination on different skin areas showed a lower contamination on breast skin than on abdominal skin by 0.4 CFU/g (*P* = 0.040). Similar to our findings Gill and Badoni (2005) also did not find any statistically significant differences in the contamination of neck skin samples and randomly selected skin samples taken after cooling with regard to TVC and *E. coli*. However, Nagel Gravning et al. (2021) found a significantly lower contamination of the breast skin compared to the neck skin samples for both the TVC and *E. coli*. It should be noted that the skin samples in their study were taken after the official meat inspection but before chilling. Comparability is therefore only possible to a limited extent, since the chilling process can have a significant influence on the bacteria adhering to the skin (Lindblad et al., 2006). Relating this data to legally required microbiological criteria is limited and only possible for *Campylobacter* spp. as there is a process hygiene criterion in place in Europe since 2018, which was recently tightened as of January 2025 (Regulation (EC) 2073/2005). The criterion requires weekly sampling of neck skin from 15 broiler carcasses after chilling. Samples are pooled to 5 groups of three neck skin. A total of 50 samples over a moving period of ten weeks is evaluated and a maximum of 10 samples is allowed to exceed 1000 cfu/g of neck skin. The sampling approach in this exploratory study did not follow the sampling scheme of the criterion, because we wanted to describe the contamination levels of individual samples instead of an arithmetic mean of pooled samples. Therefore, it is not useful to directly compare the results.

The overall mean for *Campylobacter* was 2.05 ± 0.37 log_10_ CFU/g for all sampled skin areas. A total of five samples could not be included in the analysis. The contamination of the neck skin showed no statistically significant difference to any other skin area in contrast to a study by Hutchison et al. (2019), who found significantly higher bacterial loads on neck skin than on breast skin samples. The general assumption for the higher bacterial loads on neck skin is water running down the carcass during various washing steps in the slaughter process which would result in the accumulation of bacteria on the neck skin (Mead et al., 1994).

### Comparison of neck skin and whole carcass rinse samples

**Fig. 2:**
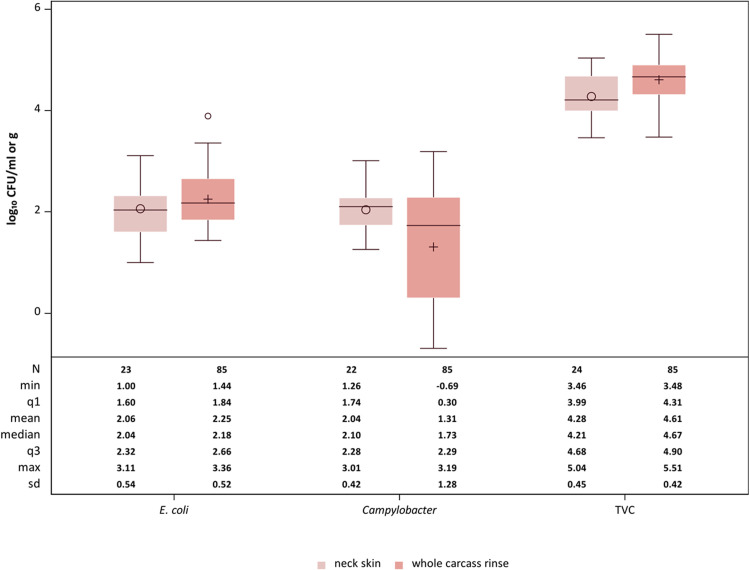
*Comparison of neck skin samples* (log_10_*CFU/g) and whole carcass rinse samples* (log_10_*CFU/ml) for TVC,* E. coli *and* Campylobacter *taken after chilling. N: number of samples, min: minimum value, q1: first quartile, q3: third quartile, max: maximum value, standard deviation.*

All data of both sampling methods could be assumed to be normally distributed (Fig. 2). For the TVC and *E. coli*, the whiskers of approximately the same length and the boxes of approximately the same size indicated homogeneity of variance. In contrast, the height of the boxes for *Campylobacter* spp. differed greatly, which is why variance heterogeneity could be assumed here. The location and dispersion measures of the neck skin and WCR samples for TVC and *E. coli* were quite close to each other, whereas the values of the WCR samples for *Campylobacter* spp. had a much higher variation than the neck skin samples (Fig. 2).

The mean values of the TVC of neck skin samples and WCR samples had a statistically significant (*P* = 0.001) mean difference of 0.33 log_10_ CFU/ml. Nonetheless, it can be said that both sampling methods provide similar results, since differences ≤ ± 0.5 log_10_ can be expected for quantitative microbiological methods of measurement in a laboratory and are therefore considered to indicate equivalence (Hübner et al., 2002). Other studies found no statistically significant difference between WCR and neck skin samples for the TVC (Gill and Badoni et al., 2005; Nagel Gravning et al., 2021). In contrast, Beterams et al. (2024b) found significantly higher counts of TVC on the neck skin samples compared to WCR.

For *E. coli* we found no statistically significant difference between both examined sampling methods (mean difference = 0.19 log_10_ CFU/ml, *P* = 0.1251). In the literature, there are studies that are in line with our results (Gill and Badoni et al., 2005) as well as those that showed a significant difference for *E. coli* loads between both methods (Beterams et al., 2024b; Nagel Gravning et al., 2021).

For *Campylobacter* spp. the calculated difference between the mean values was 0.73 log_10_ CFU/ml. The test statistic yielded a *P*-value < 0.001 and was therefore statistically significant indicating lower *Campylobacter* spp. counts in WCR samples compared the neck skin samples. These results are similar to those of Beterams et al. (2024b), while Scherer et al. (2006) found no significant difference in *Campylobacter* loads yielded by skin and rinse samples taken from packaged retail chicken thighs. Our results indicate that neck skin samples and WCR provide similar results for TVC and *E. coli*. Both sampling methods are suitable tools for process hygiene assessment. The same does not seem to be true for *Campylobacter* spp., where neck skin samples provided not only higher results but also less variation. Scherer et al. (2006) state that rinsing detaches only the slightly adherent fraction of bacteria from the skin. *Campylobacter* spp. appear to bind more tightly to the skin surface, so that mechanical tumbling in the stomacher, as is done with neck skin samples, can detach far more bacteria from the skin than shaking the rinse sample. Mechanical processing can also loosen bacteria that are located in deeper layers of the skin such as folds or feather follicles (Scherer et al., 2006). Tight attachment to the skin could be a result of the chilling process. Cooling with cold air causes the skin to dry out which results in the bacteria binding more tightly to skin surface (Lindblad et al., 2006). This effect was shown by Lindblad et al. (2006) where the prevalence of *Campylobacter* spp. was lower after the carcasses had been chilled compared to the prevalence directly before chilling.

In conclusion this study showed a similar level of bacterial contamination on the skin of broiler carcasses after chilling. Microbiological contamination on neck skin was representative for contamination levels in other areas of the carcass. Furthermore, it should be considered that sampling techniques such as destructive sampling of skin or rinsing can show significantly different results. This is important to keep in mind when comparing data reported from various studies.

## Funding

The project was supported by funds of the Federal Ministry of Food and Agriculture (BMEL) based on a decision of the Parliament of the Federal Republic of Germany via the Federal Office for Agriculture and Food (BLE) under the innovation support programme (Project No. 281C104C18)

## CRediT authorship contribution statement

**Bastian Wyink:** Conceptualization, Data curation, Writing – original draft, Writing – review & editing. **Alina Kirse:** Data curation, Formal analysis, Investigation, Writing – review & editing. **Felix Reich:** Conceptualization, Writing – review & editing, Funding acquisition, Supervision.

## Disclosures

The authors declare no competing interests in conducting the research and preparing the work for this submission.
